# Yellow Fever

**Published:** 1897-09-25

**Authors:** 


### YELLOW FEVER.

The extension of yellow fever among the Gulf towns of the United States is a matter which must cause grave anxiety in that country. For a considerable time back yellow fever has shown an increase of activity. The losses which it has caused among the Spanish troops in Cuba are known to have been very heavy, and some time ago cases were reported on both sides of the Isthmus of Panama. For several weeks past a fever has been prevailing at Ocean Springs, a watering place on the coast of Mississippi, which was at first though to be dengue; but about a fortnight ago it became manifest that the disease was yellow fever, and a rigid quarantine was established. Quarantine, however, is but a poor protection, and even before it was brought into force the infection had been carried to New Orleans. Since then cases have occurred at Scranton, and other towns in the Lower Mississippi Valley. The most serious news, however, is that it has spread to Cairo, a town a long way up the river, situated at the point where it is joined by the Ohio, and a great railway centre.

Owing to the scare, railway traffic in the affected districts has been either curtailed or suspended altogether, and quarantine is the order of the day—that pettifogging quarantine of town against town, which shows the evils and the uselessness of the system in the strongest light.

Nevertheless, we cannot be altogether surprised that people should fall back upon primitive methods in their endeavours to ward off such a disease as yellow fever. There is considerable mystery about this disease and the manner of its diffusion. It is stated with great positiveness by some that it is certainly not infectious from man to man; but what is quite clear is that it never arises outside those parts of the world where it is constantly endemic unless it is introduced by importation, and that those employed about the sick suffer very heavily. Davidson says that we have it on official authority that 22 out of 27 physicians and pharmaciens perished in the outbreak in Senegal; and the very heavy incidence of the fever on the confessing priests compared with the non-confessing priests, often residing in the same monastery, also shows the danger of intercourse with the sick.

In estimating the gravity of an outbreak of this sort one must not only bear in mind the influence of modern rapid travel in widening the area to which it can gain access before the winter checks its progress, but must also remember that we know nothing as to the causes which make a disease which is constantly within a stone's throw, so to say, of the States, only now and then at long intervals of time intrude itself upon them; nor are there any means, except the season at which it commences, by which we can foretell whether the outbreak will be virulent or not.

When occurring among susceptible people the fatality of the disease is very great, and it has caused great loss of life, not only in its special habitat but far away in the United States and in Spain. The progress of yellow fever is, however, at once stopped by frost, and it has again and again happened that when once introduced into a place it has gone on until the cold season has arrived. Little, then, as we approve of quarantine, we can well understand people flying to it who dwell in towns so situated as to be liable to attacks of yellow fever. They cannot change their climatic and physical surroundings, but they do hope to keep out the disease until the cold weather brings them safety.

